# Association between the IL-35 rs583911 polymorphism and hepatocellular carcinoma risk in patients infected with chronic hepatitis B virus

**DOI:** 10.1186/s12885-026-16609-8

**Published:** 2026-08-10

**Authors:** Hui Dong, Duo Zuo, Yuxuan Li, Zhengkun Zhu, Xia Sun, Wei Meng, Xuan Liu, Wantong Wu, Li Ren

**Affiliations:** 1 Department of Clinical Laboratory, Key Laboratory of Cancer Prevention and Therapy, Tianjin Key Laboratory of Digestive Cancer, Tianjin Medical University Cancer Institute and Hospital, National Clinical Research Center for Cancer, Tianjin’s Clinical Research Center for Cancer, Tianjin, 300060 China; 2 Department of Clinical Laboratory, Tianjin Dongli Hospital, Tianjin, 300300 China

**Keywords:** Single nucleotide polymorphism, Interleukin-35, Hepatocellular carcinoma, Hepatitis B virus, Antitumor immunity

## Abstract

**Background:**

The immunoregulatory cytokine interleukin (IL)-35 has been implicated in the pathogenesis of hepatocellular carcinoma (HCC). Here, we explored the association between single-nucleotide polymorphisms (SNPs) in the IL-35 gene and the occurrence of HCC in patients with chronic hepatitis B virus (HBV).

**Methods:**

We selected 39 patients with chronic hepatitis B (HBV) and 52 patients with HBV-related hepatocellular carcinoma (HCC) as the case groups, along with 42 healthy individuals as the control group. We genotyped five IL-35-related single-nucleotide polymorphisms (SNPs) and analyzed their association with HCC risk. In addition, serum IL-35 levels, lymphocyte counts, and regulatory T cell (Treg) frequencies were measured in peripheral blood across all groups.

**Results:**

The frequency of the rs583911 AA genotype tended to increase from healthy controls (4.8%) to patients with chronic HBV (10.3%) to patients with HCC (21.2%) (P for trend = 0.03). When patients with HCC were compared to non-HCC controls (healthy controls + chronic HBV carriers), the rs583911 AA genotype was associated with HCC risk (OR = 3.35; 95% CI: 1.17–9.61, *P* = 0.020). After adjusting for age, sex, cirrhosis status, and antiviral therapy, the association was no longer statistically significant (adjusted *P* = 0.085). Serum IL-35 levels were significantly elevated in both the HBV and the HCC groups compared with those in the control group (*P* < 0.001), with no significant difference between the HBV and the HCC groups (*P* = 0.434). Within the HCC subgroup, patients with the AA genotype tended to have higher IL-35 levels (*P* = 0.047). Flow cytometry analysis of a subset of samples (6 controls, 6 patients with HBV, and 28 patients with HCC) revealed that the number of Treg cells was greater in patients with HCC than in those with HBV (*P* = 0.038).

**Conclusion:**

Our findings suggest a potential association between the rs583911 AA genotype and HBV-related HCC risk. However, these exploratory findings did not remain statistically significant after rigorous adjustment for multiple comparisons and potential confounders; therefore, validation in larger, independent cohorts is needed.

**Supplementary Information:**

The online version contains supplementary material available at https://doi.org/10.1186/s12885-026-16609-8.

## Introduction

Hepatocellular carcinoma (HCC) is among the most prevalent and lethal malignant tumors in humans [1]. In China, the incidence of HCC is closely associated with chronic hepatitis B virus (HBV) infection [2]. The transition from HBV infection to HCC is a protracted, multistep process characterized by progressive immune dysregulation and tumor-associated immunosuppression. The hepatic immunological microenvironment plays a crucial role in the progression of HCC [3]. Accumulating evidence indicates that the upregulation of immunosuppressive inflammatory cytokines in chronic liver disease contributes significantly to the development of HCC [4–6]. Several biomarkers have been investigated to predict HCC risk in patients with chronic viral hepatitis. For instance, El-Khazragy et al. reported that elevated expression of lnc-HOTAIR could serve as a risk assessment biomarker for HCC in patients with chronic hepatitis C following antiviral therapy [7].

Interleukin-35 (IL-35), a member of the IL-12 cytokine family and a potent inhibitory cytokine [8], has been implicated in the pathogenesis of multiple autoimmune disorders, including systemic lupus erythematosus [9], rheumatoid arthritis [10], and autoimmune diabetes mellitus [11]. Notably, circulating IL-35 levels are markedly elevated in patients with chronic HBV infection, and this elevation is positively correlated with serum HBV DNA load. Emerging findings [12, 13] in tumor immunology further suggest that IL-35 contributes to tumorigenesis, particularly in hepatocarcinogenesis, by fostering an immunosuppressive tumor microenvironment. A growing body of evidence, as comprehensively reviewed by Li et al., has demonstrated that IL-35 is essential for the progression of chronic HBV infection, the development of cirrhosis, and the transformation of cirrhosis to HCC [14]. However, the dynamic changes in IL-35 expression and the potential functional impact of IL-35 gene polymorphisms during the progression from chronic HBV infection to HCC remain unclear.

Single-nucleotide polymorphism (SNP) analysis represents a powerful tool for dissecting the genetic contribution of specific genes to disease susceptibility. Previous studies [15] have linked SNPs in the IL-35-encoding genes (EBI-3 and IL12A) to the severity and progression of virus-associated liver diseases; however, their relevance to HCC development remains uninvestigated. Accordingly, the present study aimed to evaluate the association between functionally relevant IL-35 gene SNPs and HCC risk among patients with chronic HBV infection.

## Patients and methods

### Patients

A total of 133 participants were recruited from Tianjin Dongli Hospital and Tianjin Cancer Hospital between 2023 and 2024. The participants were divided into three groups: The chronic HBV group (*n* = 39), consisting of patients who tested positive for hepatitis B surface antigen (HBsAg) for more than six months; the HBV-related HCC group (*n* = 52), comprising patients with HCC and a history of chronic HBV infection; and the healthy control group (*n* = 42), consisting of healthy volunteers. The healthy controls had no family history of HCC, autoimmune diseases, or severe systemic illnesses.

The inclusion criteria for HCC patients were as follows: (1) positive for HBsAg for more than six months; (2) AFP concentration exceeding 400 ng/mL; and (3) a confirmed diagnosis of HCC via contrast-enhanced computed tomography or magnetic resonance imaging. The exclusion criteria for the HCC group included (1) coinfection with other hepatoviruses; (2) coinfection with human immunodeficiency virus; (3) the presence of autoimmune diseases; (4) the presence of other malignancies; (5) pregnancy; and (6) previous receipt of chemotherapy, radiotherapy, or immunomodulatory therapy prior to baseline sampling.

This was a retrospective case-control study. Formal sample size calculations were not performed prior to the study; the sample size was determined by the number of eligible patients available during the study period (2023–2024).

### Biochemical assays

Blood samples were collected into plain vacuum tubes without anticoagulant. After the samples were allowed to acclimate for 30 min at room temperature, they were centrifuged at 3000 rpm for 10 min. The serum was then separated and used immediately to measure liver function, kidney function, and C-reactive protein (CRP) levels using a hospital laboratory biochemical analyzer.

### Enzyme-linked immunosorbent assay

Serum IL-35 levels were measured using a commercial human IL-35 ELISA kit (ELK Biotechnology, Cat# ELK2745) according to the manufacturer’s instructions. The detection range of the kit was 15.63–1000 pg/mL, with a sensitivity of 5.8 pg/mL. Briefly, blood samples were collected into plain vacuum tubes without anticoagulant. After the samples were allowed to acclimate for 30 min at room temperature, they were centrifuged at 3000 rpm for 10 min. The serum was aliquoted and stored at -80 °C until analysis. Repeated freeze-thaw cycles were avoided.

### SNP selection and genotyping methodology for IL-35 polymorphisms

On the basis of previous studies, we selected five single-nucleotide polymorphisms (SNPs) of IL-35: rs2243115, rs568408, rs2243123, rs583911, and rs2243131. Venous blood samples were collected from each subject in purple anticoagulant tubes and stored at -20 °C until analysis. Genomic DNA was extracted from blood samples using an Ezup column blood genomic DNA extraction kit (Shanghai Shenggong Biological, Shanghai, China). Target genomic DNA fragments were amplified by polymerase chain reaction (PCR) using the primers summarized in Table 1. Amplification was followed by purification using agarose gel electrophoresis and detection via Sanger sequencing. The SNPs associated with the IL-35-encoding gene were synthesized by Shanghai BaseCraft Biotechnology Co., Ltd. The Hardy-Weinberg equilibrium (HWE) was tested in the control group for each SNP using Fisher’s exact test. All the SNPs were in HWE (*P* > 0.05), except for rs2243123, which showed borderline deviation (*P* = 0.030), likely because of the limited sample size. All the genotyping results were independently reviewed by two investigators to ensure accuracy, and the call rate for all the SNPs was 100%.

**Table 1:** Primer sequences for genotyping by Sanger sequencing

Genotyping	Primers
rs2243115	F: 5’-GGTCTTTCCTCCCAGGACTC-3’
	R: 5’-TGCCGACGTAGAGAGAGGAG-3’
rs2243123	F: 5’-TCTTGGGGAGAATCTGTTGC-3’
	R: 5’-CCACCTCCAAAGACATCCAC-3’
rs583911	F: 5’-TTTTACCCAACGGTGCTTTC-3’
	R: 5’-CTAAGCTTGGGGCATGTTTC-3’
rs2243131	F: ATGGGATCTGCACAAAGGAG-3’
	R: CTCGTCACCAAAGCAGTCAC-3’
rs568408	F: 5’-CTTTCAGAATTCGGGCAGTG-3’
	R: 5’-TTTGTTTCCACAAACACTTTGG-3’

*F* Forward, *R* reverse, PCR was performed using genomic DNA as template. The amplified products were confirmed by agarose gel electrophoresis and then sent for Sanger sequencing

### Flow cytometry assay

Fresh peripheral blood samples collected in EDTA tubes were processed within 2 h of venipuncture to minimize cell death.

TBNK cell subsets were analyzed using two ready-to-use CYTO-STAT tetraCHROME 4-color antibody cocktails (Beckman Coulter):

- CD45-FITC/CD4-RD1/CD8-ECD/CD3-PC5 (cat. no. 6607013) for T cell subsets
- CD45-FITC/CD56-RD1/CD19-ECD/CD3-PC5 (cat. no. 6607073) for NK and B cells

Regulatory T cells (Tregs) were identified using the following monoclonal antibodies (all from Beckman Coulter): CD3-ECD (clone UCHT1), CD4-PC5 (clone 13B8.2), CD25-PE (clone B1.49.9), and CD127-PC7 (clone R34.34).

The following cell populations were defined:

- CD3⁺CD4⁺ T cells: helper T cells
- CD3⁺CD8⁺ T cells: cytotoxic T cells
- CD3⁻CD19⁺ B cells: B lymphocytes
- CD3⁻CD56⁺ NK cells: natural killer cells
- CD3⁺CD4⁺CD25⁺CD127ˡᵒʷ Tregs: regulatory T cells

Compensation was performed using single-stained controls. Data were acquired on a DxFLEX flow cytometer (Beckman Coulter). The gating strategy was as follows: doublets were excluded by FSC-A/FSC-H, live cells were gated by FSC/SSC, and lymphocytes were gated by CD45. CD3⁺ T cells, CD3⁻CD19⁺ B cells, and CD3⁻CD56⁺ NK cells were subsequently identified. Tregs were identified from the CD3⁺CD4⁺ population as CD25⁺CD127ˡᵒʷ cells. A representative gating strategy is shown in Supplementary Fig. 1.

The data were analyzed using CytExpert software (Beckman Coulter).

### Statistical analysis

All the statistical analyses were performed using GraphPad Prism 8.0 (GraphPad Software, San Diego, CA, USA).

### Comparisons among groups

Comparisons among the three groups were performed using one-way analysis of variance (ANOVA) followed by Tukey’s post hoc test for normally distributed data or the Kruskal-Wallis test followed by Dunn’s post hoc test for nonnormally distributed data. Comparisons between two groups were performed using the unpaired Student’s t test or the Mann-Whitney U test, as appropriate. Categorical variables were compared using the chi-square test or Fisher’s exact test (when the expected cell count was < 5).

### Genetic association analysis

Logistic regression was used to calculate odds ratios (ORs) and 95% confidence intervals (CIs) for the association between the rs583911 AA genotype and HCC risk. Multivariate logistic regression was adjusted for age, sex, cirrhosis status (yes/no), and antiviral therapy status (yes/no).

### Comparisons of IL-35 levels

Analysis of covariance (ANCOVA) was used to compare IL-35 levels among groups, with age and sex included as covariates.

### Multiple testing correction

To account for multiple comparisons involving the five SNPs analyzed, Bonferroni correction was applied. A P value < 0.01 (0.05/5) was considered to indicate statistical significance for the SNP analyses. For all other analyses, a two-sided P value < 0.05 was considered to indicate statistical significance.

### Acknowledgements

This work was supported by the Tianjin Health Science and Technology Project (Grant No. TJWJ2023MS058) awarded to Wu Wantong.

## Results

### Distribution of IL-35 gene polymorphisms among the three study groups

The baseline demographic and clinical characteristics of the participants are summarized in Supplementary Table 1.

**Fig. 1:**
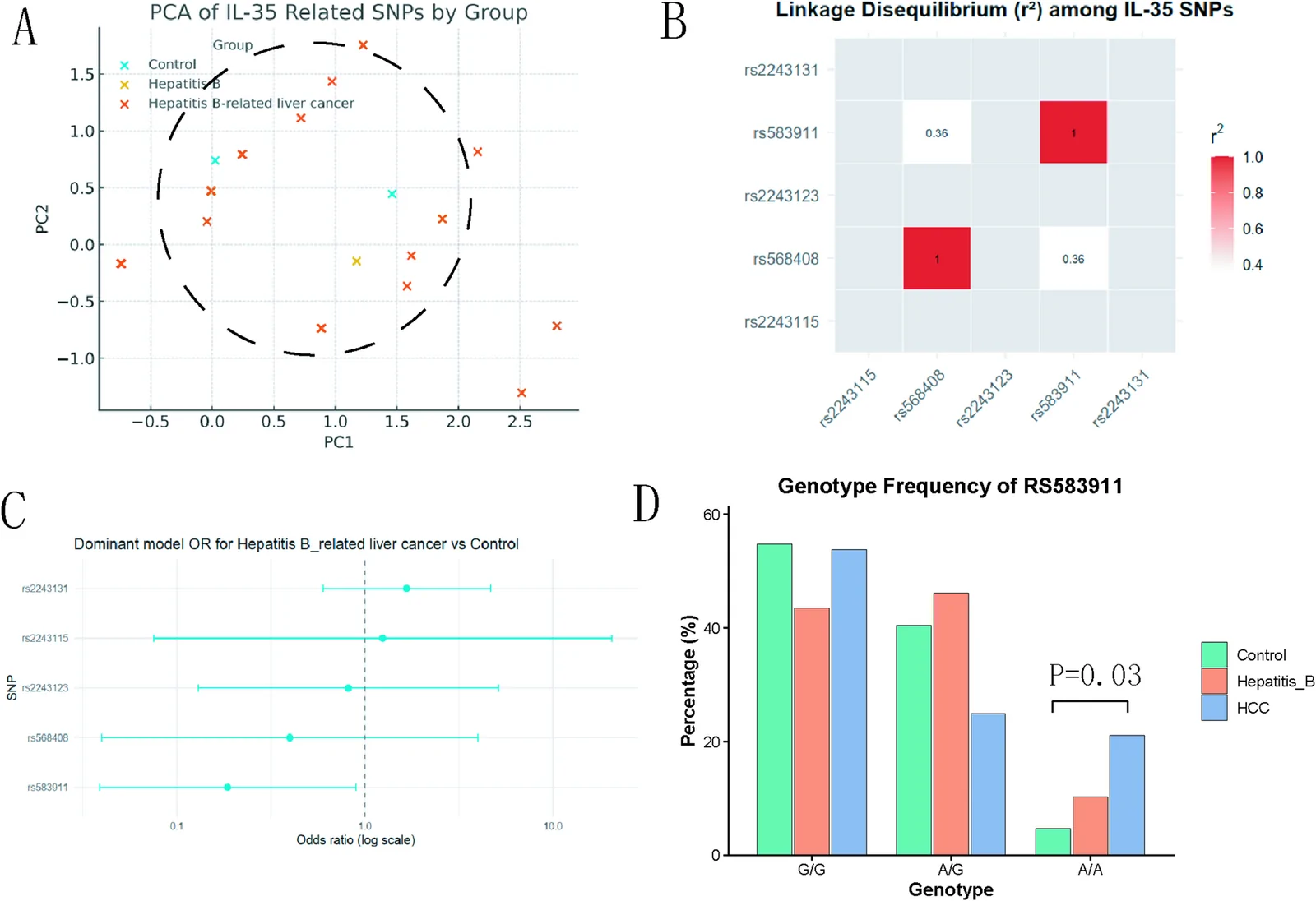
Analysis of IL-35 SNPs and their association with HCC. **(A)** Principal component analysis (PCA) plot of IL-35 SNPs among the three groups. **(B)** Linkage disequilibrium (LD) analysis of the five IL-35 SNPs.**(C)** Dominant model odds ratios for pairwise comparisons of the IL-35 SNPs. The vertical line indicates OR = 1. **(D)** Distribution of rs583911 genotypes (AA, AG, GG) among the three groups. Data are presented as means ± SD, with statistical significance defined as *P* < 0.05

We first performed principal component analysis (PCA) to evaluate the genetic variability among the three groups. The PCA plot revealed minimal clustering among the control, HBV, and HCC groups (Fig. 1A), suggesting that population stratification is unlikely to confound the association results.

Linkage disequilibrium (LD) analysis was subsequently performed to explore the genetic relationships among the SNPs. LD analysis revealed a notable correlation between rs583911 and rs568408 (Fig. 1B), suggesting that these two SNPs may be coinherited. These findings were further supported by the results of the dominant model odds ratio analysis (Fig. 1C).

Furthermore, we analyzed the genotype frequencies of the five SNPs. The frequency of the rs583911 AA genotype tended to increase from the control group (2/42, 4.8%) through the HBV group (4/39, 10.3%) to the HCC group (11/52, 21.2%) (P for trend = 0.03; Table 2). Among the five SNPs analyzed, only rs583911 showed a difference in genotype frequency among the three groups. Pairwise comparisons revealed that the AA genotype frequency was greater in the HCC group than in the control group (*P* = 0.040), whereas no significant differences were detected between the HCC and HBV groups (*P* = 0.185) or between the HBV and control groups (*P* = 0.398). (Fig. 1D; Table 2). No differences in genotype frequency were observed for the other four SNPs (rs2243115, rs2243131, rs568408, and rs2243123) among the three groups (Table 2).

**Table 2:** Genotype and allele frequencies of IL-35–related SNPs in patients among the three groups

SNPs	Genotype	Control	HBV	HCC	*P* value
*RS2243115*	G/G	1 (2.4%)	6 (15.4%)	1 (1.9%)	0.32
	T/G	6 (14.3%)	7 (17.9%)	4 (7.7%)	0.25
	T/T	35 (83.3%)	32 (82.1%)	47 (90.4%)	0.74
*RS2243131*	A/A	35 (83.3%)	30 (76.9%)	39 (75.0%)	0.65
	A/C	6 (14.3%)	8 (20.5%)	10 (19.3%)	0.71
	C/C	1 (2.4%)	1 (2.6%)	3 (5.8%)	0.31
*RS568408*	A/A	1 (2.4%)	1 (2.6%)	3 (5.8%)	0.45
	G/A	5 (11.9%)	6 (15.4%)	10 (19.2%)	0.21
	G/G	35 (83.3%)	30 (76.9%)	39 (75.0%)	0.54
*RS2243123*	C/C	2 (4.8%)	1 (2.6%)	3 (5.8%)	0.31
	T/C	4 (9.5%)	8 (20.5%)	4 (7.7%)	0.62
	T/T	36 (85.7%)	30 (76.9%)	42 (80.8%)	0.71
*RS583911*	A/A	2 (4.8%)	4 (10.3%)	11 (21.2%)	0.03
	A/G	17(40.5%)	18 (46.1%)	13 (25%)	0.09
	*G/G*	23 (54.8%)	17 (43.6%)	28 (53.8%)	0.82

Data are presented as n (%). *P* values were calculated using Chi-square test or Fisher’s exact test for genotype distribution among the three groups

HWE was tested in the control group using Fisher’s exact test (all SNPs *P* > 0.05 except rs2243123, *P* = 0.030)

### Association between the rs583911 AA genotype and HCC risk

To evaluate the potential impact of the rs583911 AA genotype on HCC susceptibility, the subjects were classified as AA genotype carriers or non-AA genotype carriers. The AA genotype frequency was higher in the patients with HCC than in the non-HCC controls (21.2% vs. 7.4%; *P* = 0.020, Fisher’s exact test). Univariate logistic regression confirmed an association between the AA genotype and HCC risk (OR = 3.35, 95% CI: 1.17–9.61, *P* = 0.020). However, after adjusting for age, sex, cirrhosis status, and antiviral therapy, this association was no longer statistically significant (adjusted *P* = 0.085). After Bonferroni correction for multiple comparisons (5 SNPs), the association remained nonsignificant (*P* > 0.05; Table 3 footnote).

**Table 3:** Association between the rs583911 AA genotype and HCC risk

	HCC (Cases)	Non-HCC Controls	Total
AA Genotype (Positive)	11	6	17
Non-AA Genotype (Negative)	41	75	116
Total	52	81	133

Non-HCC control group includes healthy controls (*n* = 42) and chronic HBV carriers (*n* = 39). OR = 3.35 (95% CI: 1.17–9.61); Univariate: *P* = 0.020 (Fisher’s exact test). Adjusted for age, sex, cirrhosis, and antiviral therapy: *P* = 0.085. Bonferroni-corrected (5 SNPs): *P* > 0.05

### Serum IL-35 levels and the correlation with the IL-35 genotype

**Fig. 2:**
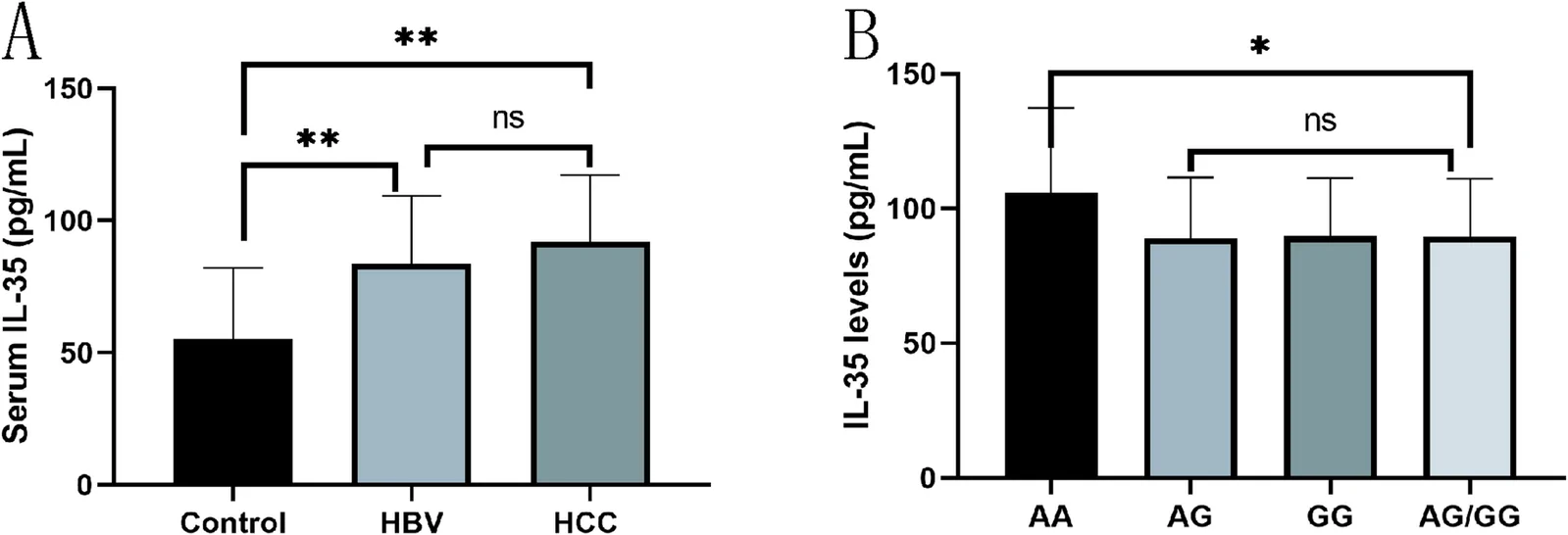
Serum IL-35 levels in the study groups. **(A)** Serum IL-35 levels measured by ELISA in the control, HBV, and HCC groups. **(B)** Serum IL-35 levels in HCC patients stratified by rs583911 genotype (AA vs. non-AA). Patients with the AA genotype showed a trend toward higher IL-35 levels than non-AA carriers (*P* = 0.047). Data are presented as mean ± SD

Serum IL-35 levels in the control, HBV, and HCC groups are summarized in Table 4. Serum levels of IL-35 were significantly greater in the HBV and HCC groups than in the control group (*P* < 0.05), and there was no significant difference between the levels in the HCC and HBV groups (Fig. 2A).

**Table 4:** Comparison of serum IL-35 levels across study groups

Group	*n*	IL-35 (Mean ± SD, pg/mL)	*P* value
Control	42	55.09 ± 27.01	— (Reference)
HBV	39	83.62 ± 25.76	< 0.001
HCC	52	92.25 ± 25.11	< 0.001

Data are presented as mean ± SD. *P* values were calculated using ANCOVA adjusted for age and sex

Given the association between the AA genotype of rs583911 and HCC risk, we further analyzed IL-35 expression levels across different genotype groups in the entire cohort. Compared with non-AA carriers, individuals carrying the AA genotype tended to have higher IL-35 levels, although this difference did not reach statistical significance (*P* = 0.088, Table 5). In patients with only HBV, no significant difference was observed (*P* = 0.587). Within the HCC subgroup, compared with non-AA carriers, patients with the AA genotype tended to have higher IL-35 levels, with a P value indicating borderline statistical significance (*P* = 0.047, Table 5; Fig. 2B). No significant associations were observed between the AA genotype or IL-35 levels and tumor differentiation or microvascular invasion in HCC patients (Supplementary Table 2).

**Table 5:** Association between rs583911 AA genotype and serum IL-35 levels in different populations

Population	AA Carriers	Non-AA Carriers	IL-35 (pg/mL)	*P* value
Entire cohort	17	116	89.70 ± 37.89 vs. 76.27 ± 28.81	0.088
HBV subgroup	4	35	76.40 ± 15.81 vs. 84.45 ± 26.69	0.587
HCC subgroup	11	41	106.10 ± 31.29 vs. 89.51 ± 21.59	0.047

Data are presented as mean ± SD. *P* value was calculated using unpaired t-test

### Treg frequency in a subset of samples

**Fig. 3:**
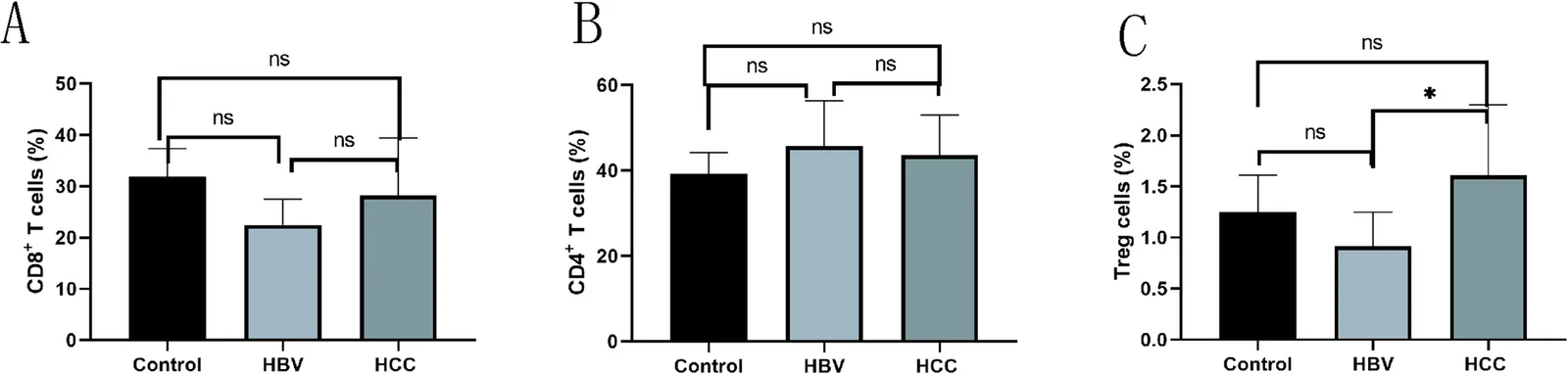
Lymphocyte subsets were analyzed by flow cytometry in a subset of samples (6 controls, 6 HBV patients, and 28 HCC patients). **A** Percentage of CD8⁺ T cells; **B** percentage of CD4⁺ T cells; **C** percentage of Treg cells. Treg frequency was higher in HCC patients (1.64% ± 0.71%) than in HBV patients (0.92% ± 0.33%) (*P* = 0.038). No significant differences were observed for other lymphocyte subsets (including CD4⁺T cells, CD8⁺T cells, B cells, and NK cells) among the three groups. Data are presented as mean ± SD

To explore the immunological basis of the IL-35-related findings, we analyzed lymphocyte subsets in a subset of samples (6 controls, 6 HBV patients, and 28 HCC patients) that were randomly selected before genotyping. The frequencies of CD4⁺ T cells and CD8⁺ T cells remained comparable among the three groups (*P* > 0.05; Fig. 3A, B). However, Treg frequency was higher in HCC patients (1.64% ± 0.71%) than in HBV patients (0.92% ± 0.33%) (*P* = 0.038; Fig. 3C). Among the 28 HCC patients analyzed, 6 carried the AA genotype (21.4%), which is consistent with the overall AA frequency in the HCC cohort (11/52, 21.2%).

## Discussion

In this study, we observed a potential association between the IL-35 SNP rs583911 and HCC risk in individuals with chronic HBV infection. When patients with HCC were compared to non-HCC controls (healthy controls + chronic HBV carriers), the AA genotype was associated with HCC risk (OR = 3.35, *P* = 0.020). However, this association was primarily driven by the comparison between patients with HCC and healthy controls (*P* = 0.040), with no statistically significant difference observed between patients with HCC and chronic HBV carriers (*P* = 0.185). Nevertheless, the increasing frequency of the AA genotype from healthy controls to chronic HBV carriers to patients with HCC (P for trend = 0.03) raises the possibility of an association, although the lack of a significant difference between HBV carriers and patients with HCC (*P* = 0.185) suggests that this trend should be interpreted with caution. Therefore, our findings do not demonstrate that the rs583911 AA genotype specifically promotes the progression from chronic HBV infection to HCC. Rather than indicating progression, these findings suggest that this variant may represent an inherent genetic susceptibility factor that precedes malignant transformation, although this hypothesis requires further investigation.

After multivariate adjustment for age, sex, cirrhosis status, and antiviral therapy, the association between the rs583911 AA genotype and HCC risk was no longer statistically significant (adjusted *P* = 0.085). This finding indicates that the observed association, while suggestive, should be considered exploratory and requires validation in larger, independent cohorts. The loss of significance after correction is likely attributable to the limited sample size, which reduces the statistical power to detect modest effects. Alternatively, the association may be confounded by cirrhosis or antiviral therapy, both of which were included in the multivariate model.

Compared with previous studies, the novelty of our work lies in its genetic perspective. Recent studies have demonstrated that IL-35 expression is elevated in HBV-related HCC tissues and promotes tumor progression through the IL-6-STAT3 signaling pathway [16] and by modulating neutrophil infiltration and CD8 + T cell function [17]. However, these studies have focused primarily on IL-35 protein expression and functional mechanisms. To our knowledge, no study has investigated the association between IL-35 gene polymorphisms and the risk of HBV-related HCC. Therefore, our study contributes new knowledge by providing the first evidence that the rs583911 polymorphism in the IL-35 gene may be associated with HCC risk in patients with chronic HBV infection. Although these findings are exploratory and require validation, they suggest a potential direction for future studies on genetic susceptibility factors for HBV-related HCC.

With respect to IL-35 levels, we found that serum IL-35 levels were elevated in both the HBV and the HCC groups compared with those in the healthy control group, but no significant difference was observed between the HBV and the HCC groups (*P* = 0.434). These findings suggest that elevated IL-35 levels may primarily reflect chronic HBV-related inflammation rather than serve as a specific marker of HCC progression.

To further investigate whether the rs583911 AA genotype influences IL-35 expression, we stratified the data by disease status. In the entire cohort, no significant difference was observed between AA and non-AA carriers (*P* = 0.088). Similarly, in the HBV subgroup, no significant difference was found (*P* = 0.587). However, the observed increase in IL-35 levels in HCC patients with the AA genotype (*P* = 0.047) suggests a potential link between the genetic variant and IL-35 expression, but these findings are of borderline significance and require further validation. Notably, the AA genotype was not associated with tumor differentiation or microvascular invasion in patients with HCC (Supplementary Table 2).

Having established the association between the AA genotype and IL-35 levels, we next investigated whether immune dysregulation is also present at the cellular level. To determine whether the observed IL-35 elevation is accompanied by cellular immune changes, we analyzed lymphocyte subsets in a randomly selected subset of samples. We observed an increase in Treg frequency in patients with HCC compared with that in HBV patients (*P* = 0.038). However, importantly, this analysis was performed on a limited subset of samples (6 controls, 6 patients with HBV, and 28 patients with HCC) because of cost constraints and the need for fresh blood processing. Therefore, these findings should be considered preliminary and hypothesis-generating. IL-35 levels were elevated in both the HBV and the HCC groups compared with those in the control group, whereas the Treg frequency was elevated only in the patients with HCC compared with that in the patients with HBV, suggesting that IL-35 may play a role in the early stage of HBV-related immune dysregulation, whereas Treg expansion may occur at the HCC stage. Although the sample size was too small to directly assess the relationship between the AA genotype and Treg frequency, the coexistence of elevated IL-35 levels and increased Tregs in HCC patients, together with the association between the AA genotype and IL-35 levels, suggests a potential link between this genetic variant, IL-35 upregulation, and Treg expansion that warrants further investigation. Further functional studies are needed to elucidate this relationship.

The role of various immune cells in the pathogenesis of HBV-related HCC has been highlighted [18]. Consistent with the findings of previous studies, Tregs play important roles in liver disease progression and the immune microenvironment in patients with HCC [19]. In the hepatic microenvironment, Tregs have been reported to protect the liver from inflammatory and autoimmune responses, while disruption of this crosstalk may result in the progression of chronic liver diseases and the development of hepatic malignancy [20].

Elevated serum and intrahepatic levels of interleukin-35 (IL-35) have been reported in HBV carriers [21]. Histopathological analyses further revealed substantial enrichment of Tregs within HCC tumor tissues, whereas IL-35 expression decreased significantly following surgical tumor resection [22]. The present study does not provide mechanistic evidence for how the rs583911 SNP affects IL-35 expression or how IL-35 regulates Treg function. Further functional studies are needed to explore the relationship between IL-35 and Tregs in the context of HBV-related HCC.

Genetic polymorphisms are well-established determinants of disease susceptibility. Previous studies have linked specific IL-35 SNPs to autoimmune disorders [23], highlighting the potential role of IL-35 in such conditions [24]. Notably, the rs583911 SNP has been associated with altered IL-35 expression and genetic susceptibility in rheumatoid arthritis [25]. In the present study, we extend these observations by suggesting a potential association between the rs583911 genotype and the risk of HCC in patients with chronic HBV infection, although these findings did not remain statistically significant after Bonferroni correction and require validation in larger cohorts.

### Limitations

This study had several limitations. First, the sample size was determined by the number of eligible patients available during the study period (2023–2024) and was relatively small, which limited statistical power and precision. This was particularly evident for the flow cytometry analysis (6 controls, 6 HBV patients, and 28 HCC patients), which was performed on a limited subset due to cost constraints. Second, the findings were not validated in an independent cohort, which limits the generalizability of the results. Third, data on smoking, alcohol consumption, antiviral therapy details, and HBV DNA levels were not available for all participants. Fourth, functional experiments are needed to establish causality between the rs583911 SNP and IL-35 expression. Fifth, although the two hospitals serve as major referral centers in North China, all participants were recruited from two hospitals in a single city, which may limit the generalizability of the findings to other populations or regions.

## Conclusions

The rs583911 AA genotype may represent a genetic susceptibility factor for HBV-related HCC, but validation in larger, well-powered cohorts is needed before definitive conclusions can be drawn.

## Supplementary Information

Supplementary Material 1.

## Data Availability

The datasets generated and/or analysed during the current study are available in the OMIX repository (https://ngdc.cncb.ac.cn/omix/release/OMIX017638) under accession number OMIX017638. The data will be made publicly available upon acceptance of the manuscript.

## Abbreviations

HCC: Hepatocellular Carcinoma
HBV: Hepatitis B Virus (HBV)
Treg: Regulatory T cell
IL-35: Interleukin-35
SNPs: Single-nucleotide Polymorphisms
