# Supplementary material for: Association between the IL-35 rs583911 polymorphism and hepatocellular carcinoma risk in patients infected with chronic hepatitis B virus
Source: BMC Cancer. 2026 Aug 10;26:1358. doi: 10.1186/s12885-026-16609-8 (PMC13737490; doi:10.1186/s12885-026-16609-8)
Supplement: Supplementary file 1 [file 12885_2026_16609_MOESM1_ESM.docx]

Supplementary Table 1. Baseline characteristics of the study participants.

| **Variable** | **Control** | **Hepatitis B** | **HCC** |
| --- | --- | --- | --- |
| Sex, n (%) |  |  |  |
| Female | 19 (45%) | 21 (54%) | 15 (29%) |
| Male | 23 (55%) | 18 (46%) | 37 (71%) |
| Age | 42.45 ± 10.67 | 46.64 ± 17.13 | 57.79 ± 9.99 |
| ALT (U/L) | 18.98 ± 8.33 | 60.26 ± 111.86 | 61.58 ± 90.75 |
| AST (U/L) | 19.57 ± 4.58 | 47.03 ± 75.19 | 67.52 ± 93.09 |
| TBIL (µmol/L) | 13.43 ± 5.31 | 18.96 ± 23.20 | 24.13 ± 51.78 |
| TP (g/L) | 72.78 ± 3.67 | 70.15 ± 6.58 | 65.48 ± 7.71 |
| ALB (g/L) | 46.81 ± 2.59 | 42.25 ± 5.99 | 40.94 ± 5.15 |
| GLU (mmol/L) | 5.13 ± 0.56 | 6.17 ± 2.39 | 5.53 ± 1.27 |
| BUN (mmol/L) | 4.32 ± 1.20 | 5.50 ± 2.71 | 4.84 ± 1.58 |
| CREA (µmol/L) | 64.98 ± 13.71 | 69.67 ± 20.21 | 68.96 ± 14.82 |
| UA (µmol/L) | 292.91 ± 91.00 | 333.12 ± 118.74 | 276.56 ± 89.44 |
| WBC (10⁹/L) | 5.83 ± 1.23 | 6.96 ± 2.77 | 6.33 ± 2.67 |
| NEU (10⁹/L) | 3.31 ± 0.99 | 4.40 ± 2.17 | 4.47 ± 2.63 |
| LYM (10⁹/L) | 1.99 ± 0.49 | 1.88 ± 0.68 | 1.29 ± 0.59 |
| MONO (10⁹/L) | 0.39 ± 0.10 | 0.53 ± 0.27 | 0.46 ± 0.22 |
| EO (10⁹/L) | 0.11 ± 0.09 | 0.12 ± 0.12 | 0.09 ± 0.10 |
| BASO (10⁹/L) | 0.03 ± 0.01 | 0.03 ± 0.02 | 0.01 ± 0.01 |
| PLT (10⁹/L) | 265.45 ± 45.40 | 189.23 ± 68.09 | 168.52 ± 74.31 |
| Cirrhosis, n(%) | 0(0) | 3(7.7) | 33(63.5) |
| Antiviral therapy, n(%) | 0(0) | 13(33.3) | 31(59.6) |

ALT, AST, and TBIL were not normally distributed; data are presented as mean ± SD for consistency.

Supplementary Figure 1. Representative gating strategy for flow cytometry analysis.

(A) Doublets were excluded by FSC-A vs. FSC-H. (B) Live cells were gated by FSC-A vs. SSC-A. (C) CD45⁺lymphocytes were gated. (D) CD3⁺T cells, CD3⁻CD19⁺B cells, and CD3⁻CD56⁺NK cells were identified. (E) Tregs were identified from the CD3⁺CD4⁺ population as CD25⁺CD127ˡᵒʷ cells.

Supplementary Table 2. Association of rs583911 AA genotype and IL-35 levels with clinicopathological features in HCC patients.

| **Characteristic** | **n** | **AA genotype (n=11)** | **Non-AA genotype (n=41)** | **P value*** | **IL-35 level (pg/mL)** | **P value**** |
| --- | --- | --- | --- | --- | --- | --- |
| Differentiation |  |  |  | 0.192 |  | 0.489 |
| Moderate |  | 3 (27.3%) | 26 (63.4%) |  | 90.50 ± 23.19 |  |
| Moderate-poor |  | 6 (54.5%) | 12 (29.3%) |  | 91.96 ± 22.13 |  |
| Poor |  | 2 (18.2%) | 3 (7.3%) |  | 106.67 ± 49.36 |  |
| Microvascular invasion |  |  |  | 0.241 |  | 0.250 |
| M0 |  | 2 (18.2%) | 23 (56.1%) |  | 85.96 ± 21.62 |  |
| M1 |  | 4 (36.4%) | 8 (19.5%) |  | 98.23 ± 23.83 |  |
| M2 |  | 5 (45.5%) | 10 (24.4%) |  | 92.43 ± 41.92 |  |

Data are presented as n (%) or mean ± SD. P values for genotype comparisons were calculated using Fisher's exact test. P values for IL-35 level comparisons were calculated using one-way ANOVA or Kruskal-Wallis test, as appropriate.
